# Extraction, Structural Characterization, and Biological Functions of *Lycium Barbarum* Polysaccharides: A Review

**DOI:** 10.3390/biom9090389

**Published:** 2019-08-21

**Authors:** Xiaojing Tian, Tisong Liang, Yuanlin Liu, Gongtao Ding, Fumei Zhang, Zhongren Ma

**Affiliations:** 1 China-Malaysia National Joint Laboratory, Biomedical Research Center, Northwest Minzu University, Lanzhou 730124, China; 2 College of Life Science and Engineering, Northwest Minzu University, Lanzhou 730124, China

**Keywords:** *Lycium barbarum* polysaccharides, structural characterization, antitumor activity, antioxidant activity, immune regulation

## Abstract

*Lycium barbarum* polysaccharides (LBPs), as bioactive compounds extracted from *L. barbarum* L. fruit, have been widely explored for their potential health properties. The extraction and structural characterization methods of LBPs were reviewed to accurately understand the extraction method and structural and biological functions of LBPs. An overview of the biological functions of LBPs, such as antioxidant function, antitumor activity, neuroprotective effects, immune regulating function, and other functions, were summarized. This review provides an overview of LBPs and a theoretical basis for further studying and extending the applications of LBPs in the fields of medicine and food.

## 1. Introduction

*Lycium barbarum* L., known as the wolfberry or goji berry, the fruits derived from *Lycium barbarum L*, are a local food that is widely distributed in the arid and semi-arid regions of China, Korea, Japan, Europe, North America, and the Mediterranean. Currently, China is the largest world producer with its 82,000 ha of cultivated land and 95,000 t of berries produced per year. The earliest use of the goji berry as a medicinal plant was at around 2300 years ago [1]. *L.* *barbarum* L. fruit is used as a traditional Chinese herbal medicine and functional food in daily life [2,3].

**Figure 1:**
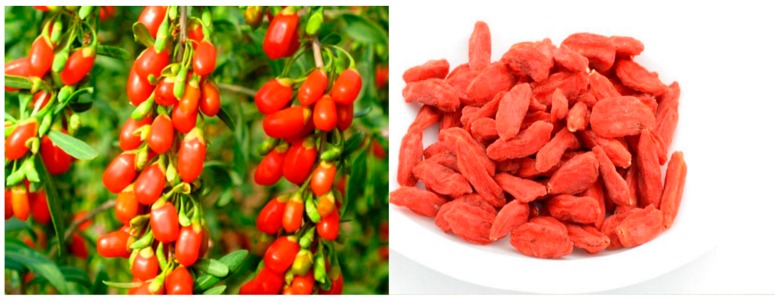
The fresh (**left**) and dried form (**right**) of *Lycium barbarum* fruit.

Three *Lycium* species (*L. barbarum*, *L. chinense*, and *L. ruthenicum*) have been discovered. Among the three species, the yield of *Lycium barbarum* is the largest in China. They are used as medicine in China and as medicinal and functional food because of their health benefits, including anti-aging, antioxidant, antidiabetic, anticancer, cytoprotective, neuroprotective, and immunomodulatory effects [4,5,6,7,8,9]. The physical appearance of *Lycium barbarum* is shown in Figure 1. The fruit is red and about 1–2 cm long. Numerous reports have been conducted to explore the function and characterization of its extracts because of the health benefits of *Lycium barbarum*. More than 200 different components, including carotenoids, phenylpropanoids, flavonoids, polyphenols, and polysaccharides, have been identified, characterized, and analyzed. Polysaccharides, vitamins, betaine, and mixed extracts of the goji berry are responsible for health benefits, such as eliciting anti-aging effects, improving eyesight, and exhibiting antifatigue effects [10].

Among *Lycium barbarum* extracts, *L*. *barbarum* polysaccharides (LBPs) isolated from *L. barbarum* fruit have been responsible for the biological activities of *Lycium barbarum*. LBPs are a group of water-soluble glycoconjugates with a molecular weight of 10–2300 kDa and comprise 5–8% of the dried fruits [11]. The beneficial health effects of LBPs, including antioxidant and antiaging effects, increased metabolism, antiglaucoma effects, immune regulation, anticancer effects, neuroprotective properties, and antidiabetic effects, have been reported [12,13,14,15,16]. According to the Chinese understanding of *Lycium* extracts and products, the content of LBPs is important for the efficacy of *L. barbarum* [17,18,19]. Therefore, as bioactive constituents of *L. barbarum*, LBPs have many biological functions to improve people’s health. The biological functions of LBPs are complex and multifaceted because of the relationship between the physiological structure and functions of LBPs. The relationship and mechanism between LBPs and human health should be fully understood. In order to give a comprehensive understanding of LBPS, the extraction methods, structure, composition, and biological functions of LBPs were summarized and discussed in this review. We collected and summarized the relative contents from previous reports to provide a theory basis for comprehensively understanding and utilizing LBPs in medical and food fields.

## 2. Extraction Methods of LBPs

**Figure 2:**
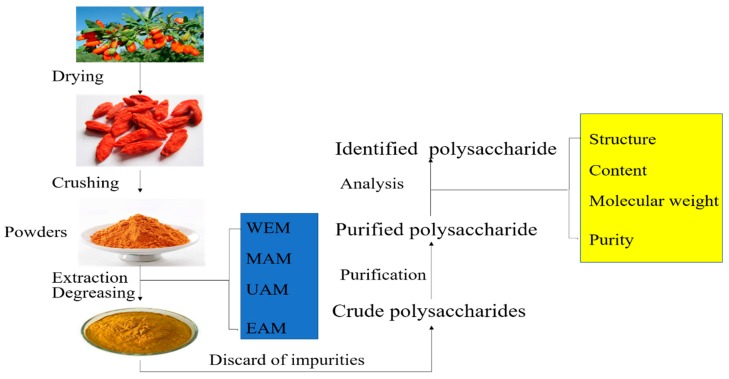
The flowchart of the extraction, purification, and analysis of *Lycium barbarum* polysaccharides (LBPs). WEM: water extraction method, EAM: enzyme-assisted extraction method, MAM: microwave-assisted extraction method, UAM: ultrasonic-assisted extraction method.

Previous reports have shown that the chemical ingredients of *L. barbarum* fruit include polysaccharides, proteins, and phenylpropanoids. Among these ingredients, LBPs account for 5–8% of the dried fruit and elicit biological effects [2]. LBPs are subjected to extraction, purification, and analysis. The flowchart of the extraction, purification, and analysis of LBPs is shown in Figure 2.

LBPs are extracted by destroying and degrading the cell wall under mild conditions without changing the properties of the polysaccharides in accordance with basic extraction principles [20]. Many LBP extraction methods, such as the water extraction method, enzyme-assisted extraction method, microwave-assisted extraction method, ultrasonic-assisted extraction method, and supercritical fluid extraction method, have been developed on the basis of this principle [21,22]. Traditional LBP extraction methods have some advantages and disadvantages. Novel LBP extraction technologies, such as ultrasound-assisted extraction method (UAE) [23,24], enzyme-assisted extraction method (EAM) [25,26], microwave-assisted extraction method (MAM) [27], and supercritical fluid extraction method (SFM) [28], have been developed to address the disadvantages of traditional extraction methods. The high extraction yield and high biological activity of LBPs are considered in choosing an extraction method [29]. 

These extraction methods have unique strengths and weaknesses [21]. Hot water extraction (HWE) is the traditional method for polysaccharide extraction. The yield of HWE is largely affected by extraction time, temperature, and the ratio of water to raw material. Also, the long duration and high temperature may lead to the degradation of the polysaccharides and decrease their biological activity. EAM possesses the advantages of environmental friendliness, high efficiency, ease of operation, and low investment cost and energy. However, the enzyme is characterized by specificity and selectivity, while several factors, such as enzyme concentration, temperature, time, and pH also affect the biological function of polysaccharides. MAM is a physical technique that is used for the extraction of polysaccharides. MAE has noticeable advantages, such as shorter extraction time, higher extraction yield, lower cost, and less solvent consumption. UAM has the advantage of improving penetration and capillary effects, leading to an increase of polysaccharides’ extractability. However, ultrasonic treatment could affect the structure and molecular weight (MW) of polysaccharides, which would cause a change in the biological activity. The extraction conditions and yield of LBP extract by different extraction methods are shown in Table 1.

As the traditional extraction method is widely used in the extraction of LBPs, the yield of the water extraction method (WEM) is 5.87% under the experimental conditions [30]. The yields of LBPs via UAM, EAM, and MAM are 2.286–5.701%, 6.81% ± 0.10%, and 8.25% ± 0.07%, respectively [31,32,33,34,35]. The yield of LBPs of novel extraction methods is higher than that of the water extraction method. Moreover, the combination of different extraction methods can obtain enhanced yields of LBPs; when the ultrasound-enhanced subcritical water extraction method (USWE) is used to extract the LBPs, high recovery yields of LBPs are produced [24].

## 3. Structure and Composition of LBPs

The separation and structural characterization methods of purified LBPs have been developed. As active ingredients, LBPs possess various biological functions. More than 33 polysaccharides have been analyzed and identified from *L. barbarum L*.

The main applied and performance techniques for the structural characterization of LBP fraction include the following techniques: (1) high-performance gel permeation chromatography (HPGPC), which is used to determine the homogeneity and molecular weight of macromolecules; (2) partial acid or enzymatic hydrolysis, oxidation with periodic acid, Smith degradation, high-performance liquid chromatography (HPLC), gas chromatography (GC), polysaccharide analysis by gel electrophoresis (PACE), and high-performance thin-layer chromatography (HPTLC). These techniques are used to determine monosaccharide composition and map the glycidic component of glycoconjugates; (3) infrared (IR) spectral analysis permits the identification of pyranosyl or furanosyl ring form and α or β anomeric configuration in monosaccharide residues; (4) ^1^H and ^13^C nuclear magnetic resonance (NMR) spectroscopy used to assign the ratios of monosaccharides present and ratios of their anomeric bonds; (5) gas chromatography–mass spectrometry (GC–MS), employed to determine the linkage positions.

Studies have widely explored the structure and composition of LBPs and demonstrated that LBPs are polysaccharides, including some ingredients of acidic heteropolysaccharides, polypeptides, or proteins [37]. The molecular weight of LBPs at the range of 10–2300 kDa. The methods used for the isolation and purification of LBPs from L. *barbarum* include DEAE ion-exchange cellulose, gel-permeation chromatography, and high-performance liquid chromatography (HPLC) [31,38]. About 20 types of polysaccharides, including Rha, Fuc, Ara, Gal, and GalA, have been investigated. Studies have shown that monosaccharide and amino acid residues constitute glycoconjugates, and, relative to glycosidic linkage analysis of glycan backbone, branching sites and side chains were considered as the structure of LBPs. The possible structure of repeat units, molecular weights, and analysis technique of previously investigated polysaccharides in L. *barbarum* are shown in Table 2.

## 4. Biological Function of LBPs

### 4.1. Antioxidant Function

Natural bioactive compounds present good biological activities, such as antioxidant, anticancer, and other functions, because of the broad diversity of structures and functionalities [54,55,56]. As bioactive compounds, LBPs have good antioxidant properties [57,58,59]. Antioxidant activity is mainly contributed by carotenoids, flavonoids, ascorbic acid and its derivatives, and polyphenols [60,61].

The biological function of LBPs have many potential functions relative to the antioxidant activity in many tissues [62,63,64]. Studies have investigated the antioxidant activity of LBPs that extract with hot water and the protective effect of LBPs against tissue oxidative injury, with results showing that LBPs exhibit a good antioxidant activity and a protective effect against skin oxidative injury [65]. The antioxidant effects of LBPs extracted by hot water have been explored via an in vivo model, showing that LBP treatments can significant increase the serum levels of SOD and GSH-Px and significantly decrease MDA contents [66,67,68,69]. The antioxidant function of LBPs shows that LBPs can significantly enhance macrophage NO, phagocytic capacity, and acid phosphatase, and exhibit good antioxidant activities in vitro [70]. LBPs can significantly increase cell viability that decreases by LPS and regulate oxidative stress by inhibiting caspase-3 activation and ROS levels in vitro [71]. Studies have investigated the effects of LBPs on stressed RPE cells and have shown that LBPs can decrease ROS levels via free radical scavenging and downstream gene function to prevent ROS-induced apoptosis [72]. Antioxidant enzyme activities, GSH levels, and MDA levels in rats fed with a high-fat diet and LBPs decreased when compared with those in the control group (*p* < 0.01) [73]. The antioxidant activity and mechanisms of LBPs are shown in Table 3.

### 4.2. Immune Regulation

Immune regulation is an important function of LBPs in people’s health. Previous studies have shown that LBPs exhibit an immune-modulating function in target dendritic cells, macrophages, T- and B-lymphocytes, and natural killer (NK) cells [79]. The immune regulation of LBPs is an important function that has been studied widely in previous studies.

Dendritic cells are used to study the effects of LBPs, and results have shown that LBPs can induce the phenotypic and functional maturation of DCs via Notch signaling and promote the cytotoxicity of DC-mediated CTLs [80]. In nutritive additives, LBPs can be used as an additive in the growth of broilers, indicating that LBPs may possess the clinical efficacy for growth promotion and immunomodulation and can be used as an alternative to nutritive additive in broilers [81]. The nanoliposome technique is used to load LBPs and study their function, showing that LBPs can significantly promote splenocyte proliferation, increase the amount of CD4(+) to CD8(+) T cells, and promote the cytokine secretion of macrophages [82]. The extract of *L. barbarum* exhibits a significant immunomodulatory activity through the promotion effects of nitric oxide and cytokines in RAW264.7 cells [83]. The efficacies of sulfated LBPs on immune enhancements in cultured chicken are high [84]. In summary, the immune regulation and mechanism of LBPs are shown in Table 4.

### 4.3. Antitumor Activity of LBPs

The antitumor activity of natural products isolated from plants have been reported [90,91,92]. Previous studies have shown that more than 100 polysaccharides exhibit good anticancer activity via in vitro studies and in vivo animal models [93].

As natural products, LBPs exhibit potential antitumor activity [94]. Studies have shown that the anticancer activity of LBPs occurs because of their effects on cancer tissue or cancer cells. The exposure to hot water extracts of *Lycium barbarum* for 24 h made cell viability reduce to 15.31% of hepatocellular carcinoma cells, as reported in a previous study [95]. Colorectal cancer is one of the most common cancers worldwide, with a study showing that the treatment of LBP (5000 mg/L) can decrease cell viability of SW480 and Caco-2 cells to 10% after 5 days of treatment, and that the treatment with LBP resulted in a dose-dependent increase in the distribution of cells in the G0/G1 phase [96]. For gastric cancer cells, LBPs can inhibit the proliferation of these cells and arrest the cell cycle at the G0/G1 phase, suggesting that LBPs are candidate anticancer agents [97]. The inhibitory effect of LBPs on the growth of glioma in rats and the underlying mechanism have been explored, and results have shown that the mechanism may be related to the regulation of the blood–brain barrier and to the promotion of CD[8]^+^ T cell invasion in the brain [98]. Studies on the effects of LBPs on the viability, cell cycle, and apoptosis of human hepatoma cells have demonstrated that LBPs can inhibit cell growth, arrest the cell cycle in the S phase, and induce apoptosis, suggesting the antiproliferative activity of LBPs by inducing cell cycle arrest and increasing intracellular calcium in the apoptotic system [99]. The anticancer activity of LBPs is mainly due to the inhibited growth of cells, arrested cell cycle, and induced cell apoptosis. The antitumor activities of LBPs on different cancer cells are shown in Table 5. LBPs exhibit a good antitumor activity on various cancer cells.

These results show that LBPs can decrease the viability of cancer cells and have inhibitory effects on cancer cells, indicating that LBPs can be used as a candidate anticancer agent in cancer treatment.

### 4.4. Neuroprotective Effects of LBPs

Natural products isolated from plants exhibit certain biological functions and have been extensively investigated because of their efficiency and biosafety. As such, they have been widely used to treat diseases. Plant extracts have been utilized to treat various functions of central nervous systems [102]. Polysaccharides are effective compounds from neurobiologically active plants, and many studies have demonstrated their beneficial effects, including neurological disorders [102,103].

The neuroprotective effects of LBPs on ischemic injury is mainly through the signaling pathways of NR2A activation and NR2B inhibition, and LBPs can be used to treat ischemic stroke [104]. A mouse experiment model of the neuroprotective effects of LBPs have shown that LBPs may exert neuroprotective effects and help prevent neurodegenerative diseases [105]. In modern society, visual impairments and blindness cause heavy damage on people’s health. LBP treatment can significantly weaken these injuries with enhanced endogenous autophagy in the body [106]. The neuroprotective effects and molecular mechanisms of LBPs are shown in Table 6.

### 4.5. Other Biological Activities

The antioxidant activity, antitumor properties, immune regulation, and neuroprotective effects of LBPs were explored in in vitro and in vivo models. Besides this, LBPs also have other biological activities, such as protecting the liver from hepatotoxicity [122], alleviating dry-eye disease [123], eliciting antidiabetic effects [124], increasing cell abilities, decreasing cell morphologic impairment, protecting against ultraviolet-induced damage [122], and alleviating CCl4-induced liver fibrosis [125]. As bioactive constituents, LBPs exhibit various biological functions and show potential benefits to people’s health. The other biological activities and mechanism of LBPs are summarized in Table 7.

## 5. Conclusions

In China, goji berries are a traditional medicinal herb that have been used for thousand years to cure disease and improve the function of the liver, kidney, and lungs. More recently, western countries have also started cultivating *Lycium barbarum* L. plants, whose fruits are consumed fresh or dried. Previous reports have showed that *L. barbarum* fruit exhibits a wide array of pharmacological activities. This beneficial *L. barbarum* component consists of a complex mixture of glycoconjugates—these being LBPs—with a molecular weight range of 10–2300 kDa, water solubility, and containing a carbohydrate portion (≥90%), represented by highly-branched polysaccharides. The LBP glycan backbones have been found to be mainly represented by α-(1→4)-galA, α-(1→6)-glc, β-(1→3)-galp (typical of arabinogalactan proteins), and β-(1→6)-galp. Other structures, less representative, are α-(1→5)-ara and β-(1→4)-galp, with different branching and terminal sites. The extraction methods of LBPs, including water extraction, enzyme-assisted extraction, microwave-assisted extraction, and ultrasonic-assisted extraction, produce different effects on the quality of LBPs. The biological functions of LBPs indicate that LBPs exhibits antioxidant, immunomodulation, antitumor, neuroprotection, and hepatoprotection.

LBPs are the main active substances in *L. barbarum* fruits and are involved in various biological functions. LBPs have great potential health benefits for further use in nutraceutical and pharmaceutical fields. The biological function of LBPs has been explored using in vitro and in vivo models but not in the human body. However, the relationship between a high-order LBP structure and bioactivities has yet to be further explored. Novel omics technologies, such as proteomics, metabolomics, and genomics, are effective in investigating the biological function of LBPs. Future works should focus on the high-order structures of LBPs, their biological function in the human body, and the relationship between their structure and bioactivity to enhance our understanding of the functional effects of LBPs. Current extraction methods give low yields and other weaknesses in the extraction of LBPs. Novel environmentally friendly and high-yielding extraction methods of LBPs should be further developed. The extraction methods and structural and biological functions of LBPs were summarized in this review to provide a useful bibliography for further investigations and applications of LBPs in medicine and food.

## Figures and Tables

**Table 1:** Summary of the extraction methods on the extraction of LBPs.

Extraction Methods	Extraction Conditions	Yield (%)	Ref
Water extraction method	The ratio liquid to solid 70:1, pH 10, at 65 °C, extracted in soakage for 3.5 h.	7.46–7.63%	[30,34,35]
Ultrasound-assisted extraction method	Extraction time of 30 min, temperature of 60 °C, solid/liquid ratio of 20 g/600 mL, power density of 300 W/L, ultrasound frequency of 28 kHz.	2.286–5.701%	[23,36]
Enzyme-assisted extraction method	Extraction time of 91 min, extraction temperature of 59.7 °C, pH 5.0.	6.81 ± 0.10 %	[26]
Microwave-assisted extraction method	Ratio of water to raw material of 31.5 mL/g, extraction time of 25.8 min, microwave power of 544.0 W.	8.25 ± 0.07%	[27]
Combination of extraction methods	Temperature of 100 °C, extraction time of 53 min, liquid-to-solid ratio of 26 mL/g, ultrasonic electric power of 160 W.	5.728%	[24]

**Table 2:** The possible structure of repeat units, molecular weights, and analysis technique of polysaccharides in *L. barbarum* [39]^.^ Definitions: SEC = size exclusive chromatography, GC–MS = gas chromatography–mass spectrometry, NMR = nuclear magnetic resonance, IR = infrared, GC = gas chromatography, ESI-MS = electrospray ionization mass spectrometry, HPGPC = high-performance gel permeation chromatography.

No	Name	Mw (kDa)	Molar Ratio	Analysis Technique	Possible Structure of Repeat Unit	Ref
1	LbGp2	68,200	Ara:Gal = 4:5	SEC, GC-MS	Backbone composed of (1→6)- β-Gal. Branches composed of (1→3)- β-Ara and (1→3)- β-Gal terminated with (1 → 3)/(1→5)- α-Ara.	[40]
2	LbGp3	92,500	Ara:Gal = 1:1	NMR	Backbone composed of (1→4)- β-Gal. Branches composed of (1→3)- β-Ara and (1→3)- α-Gal terminated with (1 → 3)/ (1→5)- α-Ara.	[41]
3	LbGp4	214,800	Ara:Gal:Rha:Glc = 1.5:2.5:0.43:0.23	NMR	Backbone composed of (1→4)- β-Gal. Branches composed of (1→3)- β-Gal terminated with (1→3)- α-Ara and (1→3)- β-Rha.	[42]
4	LBPA3	66,000	Ara:Gal = 1.2:1	Ion exchange chromatography	Heteropolysaccharide with (1→4), (1→6).	[43]
5	LBPB1	18,000	Ara:Glc = 1:3.1		Heteropolysaccharide with (1→4), (1→6) β-glycosidic bond.	
6	LBP-a4	10,200	Fuc: gal = 0.41:1	Ultrafiltration membrane method		[43]
7	LBPC2	12,000	Xyl:Rha:Man = 8.8:2.3:1		Heteropolysaccharide with (1→4), (1→6) β-glycosidic bond.	[44]
8	LBPC4	10,000	Glc	IR, GC	Heteropolysaccharide with (1→4), (1→6) α-glycosidic bond.	[45]
9	LBP1a-1	115,000	Glc		α-(1→6)- D –glucan.	[46]
10	LBP1a-2	94,000	Glc		α-(1→6)- D –glucan.	
11	LBP3a-1	103,000	GalA composed of a small amount of Gal and Ara	Gel permeation chromatography, NMR	Polygalacturonan with (1→4)- α-glycosidic bond.	
12	LBP3a-2	82,000	GalA composed of a small amount of Gal and Ara		Polygalacturonan with (1→4)- α-glycosidic bond.	
13	LBLP5-A	113,300			(1 -> 3)-linked Gal, (1 -> 4)-linked Gal, (1 -> 3)-linked Araf, (1 -> 5)-linked Araf, and (1 -> 2, 4)-linked Rhaf.	[47]
14	WSP		Rha:Fuc:Ara:Xyl:Man:Gal:Glc = 1.6:0.2:51.4:4.8:1.2:25.9:7.3	NMR, ESI-MS	Backbone composed of (1 → 2)-linked-Rha and (1→4)-linked-Gal. Branches composed of (1→5)-linked-Ara terminated with Ara residues, and (1→4)-linked-Xyl terminated with Man residues.	
15	AGP		Rha:Ara:Xyl:Gal:Glc:GalA:GlcA = 3.3:42.9:0.3:44.3:2.4:7.0	NMR	Backbone composed of linear homogalacturonan fragments and rhamnogalacturonan fragments. Side chains mainly composed of β−1,6- and β−1,4-galactopyranan and α−1,5-arabinofuranan.	[48]
16	LBP-IV	41,800	Rha:Ara:Xyl:Glc:Gal = 1.61:3.82:3.44: 7.54:1.00	DEAE-Sephadex, HPGPC, IR, UV	Backbone composed of both α- and β- anomeric configurations of Ara and Glc. Rha was located at terminal of polysaccharide chain.	[49]
17	LbGp1	49,100	Ara:Gal = 5.6:1	HPGPC	Backbone composed of (1→6)-Gal. Side chains mainly composed of (1→3)-Gal/(1→4)-Gal and (1→3)-Ara/(1→4)-Ara. Ara was located at terminal of branch.	[50]
18	p -LBP	64,000	Fuc:Rha:Ara:Gal:Glc:Xyl:GalA:GlcA = 1.00:6.44:54.84:22.98:4.05: 2.95:136.98:3.35	HPAEC-PAD, HPSEC, FT-IR, GC–MS, and NMR	Backbone composed of (1→4)- α-GalA. Side chains mainly composed of α−1,2- and α−1,4-Rha and α−1,5-Ara.	[51]
19	LBP1B-S-2	80,000	Rha:Ara:Gal:Glu = 3.13: 53.55: 39.37: 3.95	DEAE Sepharose	Backbone consisted of 1, 3-linked beta-D-Galp, 1, 6-linked beta-D-Galp and branches contained 1, 4-linked beta-D-GlcpA, T-linked beta-D-Galp, 1, 6-linked beta-D-Galp, T-linked alpha-L-Araf, T-linked beta-L-Aral 1, 5-linked alpha-L-Araf and T-linked beta-L-Rhap.	[52]
20	LRGP1	56,200	Rha:Ara:Xyl:Man:Glu:Gal = 0.65:10.71:0.33:0.67:1:10.41	HPGPC, ESI-MS	Backbone composed of (1 -> 3)-linked Gal. The branches were composed of (1 -> 5)-linked Ara, (1 -> 2)-linked Ara, (1 -> 6)-linked Gal, (1 -> 3)-linked Gal, (1 -> 4)-linked Gal and (1 -> 2,4)-linked Rha.	[53]

**Table 3:** Antioxidant activity and mechanisms of LBPs.

Antioxidant Activity	Mechanisms	Dose	Experiment Model	Experiment Type	Ref
Reduce oxidative stress	Regulating the level of MDA, SOD, GSH	100, 200, and 400 mg/kg	Rats	In vivo	[71,74]
Against hypoxia-induced injury	Down-regulation of miR-122	300 mu g/mL	Cells	In vitro	[75]
Reduces hyperoxic acute	Induced activation of Nrf2	100 mg/kg	Mice	In vivo	[59]
Attenuates diabetic testicular dysfunction	Upregulated p-PI3K and p-Akt protein expressions	40 mg/kg	Mice	In vivo	[76]
Radical scavenging	Free radical scavenging	IC 50:1.29–3.00 mg/mL(DPPH) 0.39–1.10 mg/mL (ABTS)	Chemical reagent	In vivo	[77]
Regulate the activity of enzymes	Increased activity of antioxidative enzymes	200–400 mg/kg	Rats	In vivo	[78]

**Table 4:** Immune regulation activity and mechanisms of LBPs.

Immune Regulation Activity	Mechanism	Experiment Type	Ref
Enhanced macrophage endocytic and phagocytic capacities in vivo	Activate transcription factors NFAT, AP-1, prompt CD25 expression, induce IL-2 and IFN-gamma gene transcription and protein secretion	In vitro	[85]
Regulation of immune cells	Maintain high levels of T cells, prevent the increase of Tregs, promote infiltration of CD8+ T cells	In vivo	[86]
Induce the phenotypic and functional maturation of DCs	Upregulate the expression of Notch and Jagged and Notch targets Hes1 and Hes5	In vitro	[80]
Promote the proliferation of spleen cells	Increase secretion of INF-alpha and IL-6, mRNA expression of iNOS, IL-beta and IL-6 through activating phosphorylation of ERIC, JNK, p38 and p65	In vitro	[87]
Increased immune organ indexes	Promote blood B and T lymphocyte proliferation	In vivo	[81]
Improve immune responses	Stimulate CD4(+) and CD8(+) T cell proliferation	In vitro	[88]
Enhance the immune activity	Enhance PCV2-specific IgG antibody responses, promote Th1 cytokines (IFN-gamma and TNF-alpha) and Th2 cytokine (IL-4) secretion	In vitro	[82]
Enhance the immune activity	Inhibit cell proliferation, retard cell cycle growth, and promote apoptosis	In vitro, In vivo	[89]

**Table 5:** Antitumor activities and mechanism of LBPs.

Antitumor Activity	Mechanism	Tumor Model	Experiment Type	Ref
Reduce cell viability	Inhibit growth of tumor	MCF-7, T47D, SMMC-7721, DU145	In vitro	[7,44,100]
Regulate apoptosis	Induce apoptosis	MCF-7, BIU87	In vitro	[100]
Regulate cell cycle	Arrest the cells at the G1 phase	SW480, Caco-2 cells	In vitro	[101]
Regulate immune activity	Enhance immunity	Mice	In vivo	[84,86]

**Table 6:** Neuroprotective effects and mechanisms of LBPs.

Neuroprotection Effects	Molecular Mechanism	Experiment Type	Ref
Improve neurodegenerative diseases	Increase the activity of Akt; regulate the expression of HSP60/HSP70; reduce caspase cascade reaction	In vitro, In vivo	[107,108,109,110,111]
Inhibition of oxidative stress	Increase SOD, CAT and GSH-Px; decrease the ROS level, inhibit JNK pathway	In vitro, In vivo	[112,113,114,115]
Inhibition of inflammation	Inhibit of NF-κB	In vivo	[116]
Inhibit abnormal differentiation of nerve cells	Increase differentiation of hippocampal neuron stem cells and inhibit abnormal differentiation	In vitro	[117]
Inhibition of apoptosis	Promotes Bcl-2, inhibits Bax, overexpression of CytC gene	In vivo	[118]
Reduce glutamate toxicity	Decrease neurotoxic effects of glutamate on PC12 cells; inhibition of ROS accumulation, LDH release and Ca[2]^+^ overload	In vivo	[119,120]
Inhibit the tube formation of microvascular endothelial cells	No report	In vivo	[52]
Neuroprotective agent in ischaemic retinopathies	Enhance immunoreactivity of protein kinase C alpha and attenuated glial fibrillary acidic protein expression	In vivo	[121]^.^

**Table 7:** Other biological activities and mechanism of LBPs.

Biological activities	Mechanism	Experiment type	Ref
Attenuates diabetic testicular dysfunction	Inhibition of the PI3K/Akt pathway-mediated abnormal autophagy	In vivo	[76]
Inhibit the vascular lesions	Regulating p38MAPK signaling pathways, inhibiting absorption of glucose	In vivo	[124,126]
Prevents against ultraviolet-induced damage	Activation of Nrf2	In vivo	[122]
Protect the liver from hepatotoxicity	Regulating oxidative stress	In vivo	[62]
Alleviating effects of CCl4-induced liver fibrosis	Inhibition of the TLRs/NF-kappa B signaling pathway expression	In vivo	[125]
Alleviating dry-eye disease	Schirmer’s test, tear break-up time (BUT) measurement	In vivo	[123]
Protects against neurotoxicity	Upregulating Nrf2/HO-1 signaling	In vitro	[127]
Ameliorate Cd testicular damage	Regulate oxidative stress	In vivo	[128]

## Abbreviations

LBPs	Lycium barbarum polysaccharides
WEM	water extraction method
EAM	enzyme-assisted extraction method
MAM	microwave-assisted extraction method
UAM	ultrasonic-assisted extraction method
SEC	size exclusive chromatography
ESI-MS	electrospray ionization mass spectrometry
GC–MS	gas chromatography–mass spectrometry
NMR	nuclear magnetic resonance
HPGPC	high performance gel permeation chromatography
